# Who Benefits Most from the Family Education and Support Program in Cape Verde? A Cluster Analysis

**DOI:** 10.3390/children11070782

**Published:** 2024-06-27

**Authors:** Adriana Correia, Cátia Martins, Rita dos Santos, Victoria Hidalgo, Saúl Neves de Jesus, Cristina Nunes

**Affiliations:** 1 Research Centre for Tourism, Sustainability and Well-Being (CinTurs), University of Algarve, Campus de Gambelas, 8005-139 Faro, Portugal; a62613@ualg.pt (A.C.); snjesus@ualg.pt (S.N.d.J.); 2 Psychology Research Centre (CIP), University of Algarve, 8005-135 Faro, Portugal; csmartins@ualg.pt (C.M.); rasantos@ualg.pt (R.d.S.); 3 University Research Center in Psychology (CUIP), University of Algarve, Campus de Gambelas, 8005-139 Faro, Portugal; 4 Department of Developmental and Educational Psychology, Faculty of Psychology, University of Seville, 41018 Seville, Spain; victoria@us.es

**Keywords:** family education and support program (FAF), parental attitudes and beliefs, parental educational practices, perceived parental efficacy, positive parenting

## Abstract

Background/Objectives: Child parenting programs can enhance parental skills, prevent future issues in child development, and improve children’s quality of life. The present research aimed to study the changes promoted by the Family Education and Support Program (FAF) implemented in Cape Verde, regarding parental educational practices, perceived parental efficacy, and attitudes and beliefs of Cape Verdean parents. Methods: To this end, 37 participants were evaluated through a pretest-postest design. The evaluated dimensions were perceived parental competence, parenting practices, Parental attitudes and beliefs, mental health and perceived child quality of life. Results: A cluster analysis was conducted, distinguishing two groups. Both groups benefited from the program. Cluster 1 reported more significant gains in dimensions of parental efficacy and satisfaction, inadequate expectations, affection and support, and reactivity, while cluster 2 showed a greater difference in regulation and reactivity. Conclusions: Overall, the FAF intervention contributed to an increase in positive parenting practices. By analyzing potential underlying profiles in the change process, this study suggests that there are participants who benefit more than others from the intervention, and this information may be relevant for professionals and researchers in the field.

## 1. Introduction

Parenting can be defined as the interactions, emotions, beliefs, attitudes, practices, knowledge, and behaviors of parents that are associated with providing comprehensive care to their children. It refers to the ongoing process of promoting and supporting the full development and socialization of the child. Among the many influences that impact child development, parents are essential for the development, protection, empowerment, adaptation, and success throughout the lives of children. Children who are educated with strategies involving attention, care, and stimulation become more cooperative, react more positively to non-punitive approaches, and demonstrate greater flexibility and adaptability [1,2].

Parenting programs, which promote positive interactions between parents and children, can prevent future issues in child development, strengthen emotional bonds, and consequently protect children from violence, as well as promote their overall health [3,4,5,6]. The essential components of parenting programs include education and counseling for parents and caregivers on positive parenting practices, such as the use of non-violent discipline, and effective and sensitive communication strategies to deal with children and adolescents [7,8,9,10]. By increasing parents’ knowledge about child development, improving parenting skills, and encouraging the use of positive strategies in child education, these programs can prevent the use of negative parenting practices and various forms of violence [11,12,13,14].

Positive parenting refers to respectful, caring, protective, nurturing, and affectionate parental behaviors that stimulate and promote the satisfaction of children’s basic needs. It involves providing guidance and setting boundaries to strengthen the child’s full development [15]. In contrast, negative parenting is characterized by rigid and coercive, punitive, and violent practices [16,17,18].

Parental practices are strategies adopted to suppress inappropriate behaviors and promote appropriate behaviors [19,20]. They guide, teach, and correct children’s attitudes and behaviors and can be either positive (e.g., positive discipline and parental involvement in interactions with children) or negative (e.g., physical and psychological abuse, neglect, negative communication) [20,21]. Parents should combine affection and protection while establishing educational boundaries, as opposed to the undesirable use of physical and psychological punishments, which induce feelings of depreciation, fear, behavioral alterations, and emotional dysregulation in children [22].

Many factors influence parenthood, including evolution and history, culture, socioeconomic level, family typology, number of children, children’s ages, and parents’ mental health and social support [16,23,24]. The choice of a particular parental practice can also be influenced by the individual characteristics of both parents (e.g., developmental history, personality, psychopathology) and children (e.g., temperament), as well as by the sociocultural context in which they are a part [20,25]. According to Belsky [26], it is crucial to highlight the developmental history of the individual, as it influences their personality, and consequently has implications for their parental practices. In many cases, parents who experienced adverse situations in childhood (e.g., physical punishment) often repeat these practices with their children, perpetuating an intergenerational cycle of violence [18]. Another important aspect concerns the use of violent disciplinary practices, which, instead of being a deliberate choice of discipline, result from parents’ anger and frustration or lack of knowledge about the harms of violence and the possibility of using non-violent practices [27,28].

The relationship between parental practices and various dimensions of child well-being has also been extensively studied, highlighting the influence of parental attitudes and behaviors on children’s psychosocial adjustment, academic performance, and health [29,30,31]. While psychological control and coercion have been associated with psychosocial maladjustment in children, behavioral control and positive parenting have been related to healthy development [32,33,34]. A pattern of punitive parental behavior (e.g., hitting, threatening, and scolding) is associated with long-term risk trajectories for both externalizing and internalizing problems [35,36]. In this regard, parents who employ harsh discipline, including physical punishment with low responsiveness and inconsistency, are associated with adolescents experiencing more health problems such as substance abuse, mental health issues, disengagement, and school dropout [37,38]. Conversely, positive involvement between parents and children has reduced the impact of coercion, decreasing children’s behavioral problems [39,40,41].

Parenting has been extensively studied, assessing the relevance of cultural context in the effectiveness of certain parenting behaviors [20]. Some studies have shown that a high level of parental control may have positive effects for African American and Asian American youths [42,43], but not for European American youths. The cultural and socioeconomic aspects of parents (e.g., educational level and professional qualifications) have a moderating effect on parenting practices, affecting children’s psychosocial adjustment and well-being. These differences found across cultures suggest that parenting practices have different meanings and implications depending on the sociocultural context [20].

Regarding parental attitudes, they involve a set of beliefs that position parents regarding a particular issue or decision-making process (e.g., being against or in favor of corporal punishment as an educational strategy) [44]. In studies conducted on parental attitudes, especially in abusive parents, it was observed that they tend to have inappropriate expectations about children’s abilities, associated with a lack of knowledge about their needs at different developmental stages [45] and a lack of empathy toward the child [46]. Parte superior do formulário On the other hand, abusive parents typically have a negative self-image, based on experiences of exposure to ridicule, disappointment, and failure during their own childhoods, which they tend to replicate, thereby extending this negative view to their children. The behaviors exhibited by children, which abusive parents believe should be eliminated, often mirror those for which they themselves were punished as children, thus imbuing corporal punishment with a sense of approval and tradition. The outcome of such abusive behavior is the development of aggressive behaviors in the child [47].

The parents’ perception of self-efficacy—which translates into their expectations regarding the adequacy of their parenting skills in prioritizing the child’s needs according to their level of development [48]—is also related to the parenting practices used. Thus, parents with high perceived self-efficacy use more positive parenting practices, while parents with low self-efficacy have limited abilities to effectively deal with challenging children, opting to give up or use punitive and severe strategies [49]. Therefore, the perception of parental competence is even more important in families at psychosocial risk, where the exercise of parenting can be more challenging [1,24,50,51]. Parents’ sense of competence consists of the parents’ beliefs about their ability to influence the development of their children in a positive way and the satisfaction derived from the parental role [48,49,50,51]. Parental satisfaction includes attitudes towards children, the nature of your relationship with them and the attitudes given the responsibilities inherent to the parental role [9,49].

When parental satisfaction and perception of parental self-efficacy are compromised, high levels of parental stress may arise, resulting in symptomatic consequences at cognitive, emotional, and behavioral levels (e.g., anxiety, depression, post-traumatic stress, obsessive-compulsive thoughts, and somatic complaints) [52]. Therefore, parental stress influences parental well-being, which arises from the conflict between demands and available resources, generating negative feelings about oneself and the child and influencing how parents act. The higher the level of parental stress, the worse the environment provided for the children, as they become more vulnerable to anxiety and externalizing problems [50,53,54]. Parental stress can thus result in low parental satisfaction, especially when there is weak family and social support, a low perceived quality of life, and psychological problems [50,54].

Regarding the influence of parenting practices on parental mental health, results have shown that engaging in positive parenting (i.e., more practices of emotional support and fewer practices of rejection and control) is associated with higher levels of perceived psychological quality of life in adults [55]. This finding underscores that more positive relationships are associated with higher levels of life satisfaction, particularly in the family context, influencing the family environment and contributing to individual well-being [1]. In this sense, there is a need to address not only the needs of children but also the needs of parents, as their psychological well-being is crucial for good parenting practices [56].

Strategies for dealing with stress primarily focus on valuing interpersonal relationships, balancing work and leisure, and maintaining healthcare, so participating in parental intervention programs can be a useful tool because parents can find and increase the necessary social support in this context [24,50]. Thus, the implementation of intervention programs based on positive parenting becomes even more important, especially in contexts that are inherently more likely to generate parental stress, such as in developing countries, where economic difficulties, overcrowding, and low qualifications and literacy lead to environments of greater problems in terms of family functioning, including conflict situations and negative parenting practices [57], as is the case of Cape Verde [58].

In Cape Verde, and more specifically on Boa Vista Island (where the FAF took place), data on living conditions demonstrate very precarious situations, with few resources, overcrowding and poverty, which contribute to the increase in situations of hostility and of conflicting interactions between the couple and their children. Children are exposed to child labor, sexual abuse and mistreatment, with 57% of children aged zero to six being physically punished by caregivers for disobedience. Parents’ working conditions are very demanding, which can make it difficult to organize and structure activities with their children, which can result in emotional distance and a lack of parental support [58]. Study of the implementation of positive parenting programs in Africa has been rare, and non-existent in Cape Verde [58]. Several programs promote positive parenting (e.g., Triple P; Sanders [59]; Incredible Years; Gardner et al. [60]; Learning Together, Growing With Family; Amorós-Martí et al. [61]), some of which have been implemented in African contexts [62], albeit in English. The Family Education and Support Program (FAF) [63,64,65] is an evidence-based program that focuses on positive parenting with a psychoeducational and community-based approach, employing participatory and experiential methodology to enhance parenting skills. This program has been implemented in various countries, particularly low- and middle-income (LMI) countries, with different cultural and socioeconomic backgrounds, with a special emphasis on families at psychosocial risk (e.g., Correia et al. [12]; Hidalgo et al. [66]; Maya and Hidalgo [67]). The main objectives of this program are: (a) to improve parenting practices used by parents; (b) to strengthen feelings of security in their role as parents, enhancing parental competence; (c) to promote community integration of families; and (d) to improve the quality of life for parents and children [64].

The topics covered and the activities carried out were those contained in the FAF program manual (Hidalgo et al., [64]), selected according to the characteristics of the participants and the identified intervention needs, including child development, adolescent development, adult development, family system, educational styles (norms and discipline; affection and communication), conflict resolution, risky sexual behaviors and substance use. The FAF was applied in 12 sessions (on a weekly basis, 2 h per session) by two psychologists, with specific training for this program.

Considering that the FAF intervention had already been translated and adapted for the Portuguese context [68], it was decided to implement the program in the African context, specifically in Cape Verde. Most of the research conducted in African contexts has focused more on the effectiveness of programs [12,69,70,71,72], although some investigations in the field of positive parenting promotion had already focused more on participant profiles and underlying characteristics of the change processes [73,74]. Thus, the present research aimed to study the changes promoted by the FAF in parenting practices, perceived parental efficacy, and parents’ attitudes and beliefs. Namely, we intend to identify which parents of this group most benefit from the intervention and their characteristics.

## 2. Methods

### 2.1. Participants

A total of 35 mothers (94.6%) and 2 fathers (5.4%) participated in this study, ranging in age from 25 to 56 years (*M* = 35.62; *SD* = 7.23). Regarding their educational qualifications, 45.9% had incomplete primary education, 29.7% had incomplete secondary education, and 16.2% had a higher education degree. Overall, most participants were professionally active (91.9%), engaged in low-skilled (56.8%) or medium-skilled jobs (27%), with job stability (86.5%) and incomes ranging from 4.000 to 75.000 Cape Verdean escudos (*M* = 30,366.67; *SD* = 17,991.35). Regarding family structure, 70.3% were biparental families and 21.6% were monoparental families, with 54.1% being nuclear families and 37.8% being reconstituted families, with 69.4% reporting family stability. Regarding the children, 45.9% of the families had female children and 51.1% had male children with ages ranging from 6 to 12 years (*M* = 8.68; *SD* = 2.27). In terms of past psychosocial risk, 51.4% had no risk, 32.4% had level 1 risk, 10.8% had level 2 risk, and 5.4% had level 3 risk; for current risk, 56.8% had no risk, 24.3% indicated level 1, and 10.8% indicated level 3.

### 2.2. Measures

*Sociodemographic data.* Participants responded to an interview-format questionnaire consisting of 13 items that assessed individual indicators (age, origin, level of education, and professional status) and family indicators (family type and size, family stability, number of children, and income).

Level of psychosocial risk. The Inventory of Stressful and Risk Life Events (Hidalgo et al. [75]; translated by Nunes et al. [76]) was used to assess the level of psychosocial risk, consisting of a list of stressful and negative events (e.g., “Conflictual relationship with children” or “Being a victim of abuse”), which can characterize both past life trajectories (i.e., 7 items: “Childhood maltreatment”) and the present situation of the individual (i.e., 15 items: “Being a victim of maltreatment”). Higher scores indicate a higher level of associated risk.

Perceived parental competencies. The Portuguese version of the Parental Sense of Competence (PSOC) (Johnston and Mash [77]; adapted by Nunes et al. [5]) consists of 16 items that evaluate parental competence perceived by parents across two dimensions: efficacy (i.e., 7 items: e.g., “Even though it is difficult, I already know how to influence my children”; α = 0.70) and satisfaction with the parental role (i.e., 9 items: e.g., “Being a mother makes me feel nervous and anxious”; α = 0.72), measured on a scale from 1 to 6 (1 = “No, I totally disagree” and 6 = “Yes, I totally agree”). Higher scores indicate higher levels of efficacy or satisfaction. According to the extensive review of Jones & Prinz [49], the PSOC scale is the most frequently used tool in assessing parenting self-evaluations.

Mental health. The General Health Questionnaire (GHQ-28; Goldberg and Williams [78]; Portuguese adaptation by Pais-Ribeiro and Antunes [79]) was used to assess non-psychotic psychiatric disorders. The GHQ-28 is self-report screening. measure used to detect possible psychological disorder and identifies two main concerns: the inability to carry out normal functions; and the appearance of new and distressing phenomena [78]. It consists of 28 items (e.g., “Have you been feeling perfectly well?”), answered on a Likert-type scale from 0 to 3 (0 = “Not at all” and 3 = “Much”). The total score of the questionnaire varies between 0 and 84, with higher values indicating poorer mental health (α = 0.91).

Perceived child quality of life. This was measured using the Kidscreen-10 (The European Kidscreen Group [80]; Portuguese version by Gaspar and Matos [81]), a scale composed of 10 items (e.g., “Think about the last week … did your child feel lonely?”) that assesses the well-being and subjective health of the child on a 5-point scale, ranging from 1 = “Not at all” to 5 = “Completely”. Higher values correspond to a better perceived quality of life (α = 0.74). The KIDSCREEN-10 is recommended by the International Consortium for Health Outcomes Measurement as part of their standard set of outcome measures for anxiety disorders, depression, obsessive-compulsive behavior disorders, and post-traumatic stress disorder in children and adolescents [80].

Parenting practices. This was an instrument composed of a compilation of subscales from various instruments that assess different aspects related to parental behavior: affection and support, reasoning/induction, democratic participation, permissiveness, excessive reactivity, and intrusion. The Affection and Support (AP), Regulation (RE), and Autonomy (AU) scales are specific subscales of the short version of the Parenting Styles and Dimensions Questionnaire-13 (Robinson et al. [82]; Portuguese adaptation by Martins et al. [19]). For each item, participants indicate the frequency with which they perform the presented behaviors using a 5-point Likert scale (1 = “Never” to 5 = “Always”). The democratic style includes subscales of Support and Affection (5 items; e.g., “I praise my child when they behave or do something well”; α = 0.69), Regulation (5 items; e.g., “I emphasize the reasons for the rules I establish”; α = 0.77), and Autonomy/Cedence of Autonomy/Democratic Participation (5 items; e.g., “I encourage my child to express themselves freely, even when they disagree with me”; α = 0.68). Responses are given using a 5-point Likert scale (1 = “Never” to 5 = “Always”), and higher scores on each scale reflect more frequent use of each practice. Excessive Reactivity (ER; 5 items; e.g., “When my child misbehaves, I raise my voice or shout”; α = 0.68) evaluates the tendency to respond impulsively to children’s misbehavior (ER), and Permissiveness evaluates the lack of parental control (5 items; “I let my child do whatever they want”; α = 0.57); both are subscales of the reduced version of the Parenting Scale-15 [83]. Items are responded to using a 7-point Likert scale (1 = “Never” to 7 = “Always”). Low scores indicate good parenting. Intrusion is a subscale of the Psychological Control Scale (Barber [84]; Portuguese version by Nunes et al. [20]), composed of 8 items (e.g., “My father/mother tries to make me change my mind”; α = 0.57), which assesses the extent to which parents try to intrusively control their children. Items are responded to on a 6-point scale (1 = “Strongly disagree” to 6 = “Strongly agree”), and higher scores reflect more frequent use of this practice.

Parental attitudes and beliefs. We used two subscales from the Adult Adolescent Parenting Inventory (AAPI; Bavolek and Keene [47]; Portuguese version by Lopes and Brandão [85]) to assess parental educational attitudes. The Inappropriate Expectations subscale (7 items: e.g., “Good children always obey their parents”; α = 0.77) evaluates the extent to which parents have a realistic perception of children’s development, abilities, and limitations. The Physical Punishment subscale (11 items: e.g., “Spanking children when they misbehave teaches them how to behave”; α = 0.78) assesses the extent to which parents value or do not value physical punishment as a means of disciplining and educating their children. Higher scores indicate favorable parental attitudes and behaviors, while lower scores indicate a greater potential for abuse or neglect.

### 2.3. Analysis Plan

The data were entered into SPSS 29.0.1.0 (IBM Corp., Chicago, IL, USA) and cluster analysis was conducted based on the on clValid [86] an *R* package for cluster validation [87]. This package contains several methods for validating the results from a cluster analysis. Multiple clustering algorithms, validation measures, and numbers of clusters were used simultaneously, to determine the most appropriate method and an optimal number of clusters for the dataset. The optimal scores, along with the corresponding cluster method and number of clusters, were extract for the clustering results of a particular algorithm. The validation measures used included internal measures [88], namely, the Dunn Index [89], Silhouette Width [90] and the connectivity (where scores near 0 are preferable), and included stability measures, namely, the average proportion of non-overlap (APN), the average distance (AD), the average distance between means (ADM), and the figure of merit (FOM) [91]. The Silhouette value measures the degree of confidence in the clustering assignment of a particular observation, with well-clustered observations having values near 1 and poorly clustered observations having values near −1. Together with the Dunn Index (the ratio of the smallest distance between observations not in the same cluster to the largest intra-cluster distance), are examples of non-linear combinations of the compactness and separation. Connectivity is another internal measure available in clValid and refers to the more related and nearby observations [91].

The order of clustering algorithms on each validation measure is rarely the same; therefore, to select the cluster algorithm, a rank aggregation was used as it ranks all the clustering algorithms based on their performance, determined by all the validation measures simultaneously, and also determines the overall winner. The rank aggregation was performed using the *R* package RankAggreg.

The internal consistency levels of the measures used were calculated using Cronbach’s alpha, with values between 0.60 and 0.70 considered satisfactory, above 0.70 as adequate, and above 0.90 as excellent [92]. To analyze the characteristics of the participants in each cluster, descriptive statistics (mean, standard deviation, minimum and maximum values), as well as frequencies (f) and percentages (%) for categorical variables, were used. Considering the characteristics of the clusters, the non-parametric Mann-Whitney test was used to compare mean values between the two groups, with differences considered significant when *p* < 0.05 (α = 5%). Effect sizes were calculated using the *r* value, with values between 0.20 and 0.40 considered of low magnitude, between 0.40 and 0.60 of moderate magnitude, between 0.60 and 0.80 high, and above 0.80 as very high [93].

## 3. Results

**Figure 1:**
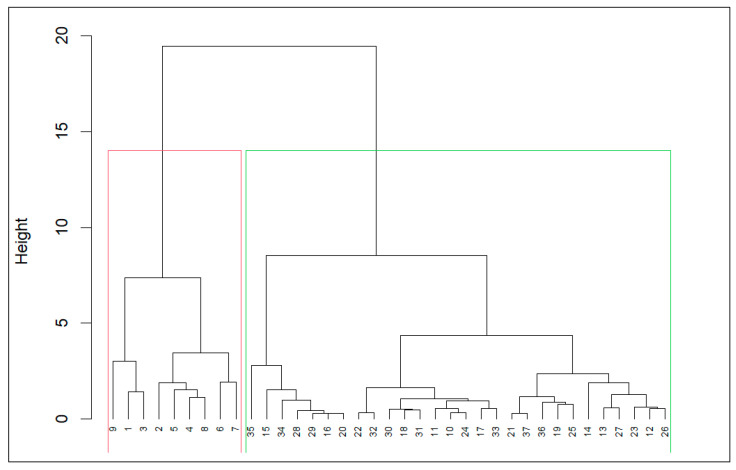
Dendrogram with the 8 variables under analysis.

The choice for the best performing algorithm was not straightforward in this case because the order of the clustering on each validation measure is not the same (Table 1). Therefore, a rank aggregation was performed using the default cross-entropy method with weighted Spearman’s footrule to produce a 4-optimal order (i.e., hierarchical–2, sota-3, diana-3, diana-2; Spearman = 4.309), pointing that an hierarchical solution with 2 clusters was the best performing algorithm for this case (Figure 1).

According to Table 2, cluster 1 (C1) consisted of 9 participants, all mothers (100% female) aged between 26 and 56 years (*M*_C1_ = 36.78; *SD_C_*_1_ = 8.01), predominantly attending group 3 (88.90%). Cluster 2 (C2) showed greater heterogeneity, consisting of 29 participants, mostly mothers (85.70%), followed by fathers (7.10%) and grandparents (7.10%), with 85.70% being female, aged between 25 and 54 years (*M*_C2_ = 35.25; *SD*_C2_ = 7.74), distributed across assistance groups 2 (35.70%), 3 (28.60%), and 4 (32.10%).

Per the participants’ educational level, in C1, 44.40% reported incomplete primary education and 33.30% had completed secondary education. In C2, 46.40% had incomplete primary education, 28.60% had secondary education, and 17.90% had higher education. Regarding employment, in C1, 100% were active but did not demonstrate stability (88.90%) and performed low/no qualification (55.60%) or medium qualification jobs (33.30%). In C2, 89.30% were active, with stability (85.70%), and performed low/no qualification (57.10%) or medium qualification jobs (25.00%).

Regarding family characteristics, both C1 and C2 were composed of biparental families (C1 = 66.70%; C2 = 71.40%), nuclear families (C1 = 55.60%; C2 = 53.60%), with family stability (C1 = 55.60%; C2 = 71.40%). However, C1 families lived with fewer people than C2 families (*M*_C1_ = 3.89; *SD*_C1_ = 1.62; *Range*_C1_ = 2–6; *M*_C2_ = 4.89; *SD*_C2_ = 1.20; *Range*_C2_ = 3–8). The incomes of C1 families ranged between 1,000,000 and 50,000 escudos (*Range* = 11.20–22.20%), while in C2 families they were mostly between 10,000 and 30,000 escudos (25%). Regarding children, in both clusters the age ranged from 6 to 12 years (*M*_C1_ = 8.33; *SD*_C1_ = 2.50; *M*_C2_ = 8.79; *SD*_C2_ = 2.23), with C1 children being predominantly male (66.70%), while the C2 children were equally of both genders (50%).

Regarding past psychosocial risk, participants in C1 reported between 0 and 2 events (*M* = 0.67; *SD* = 0.87), and those in C2 experienced between 0 and 3 events (*M* = 0.71; *SD* = 0.90). At the level of current risk, the C1 participants reported a number of stressful life events (*M* = 1.00; *SD* = 1.00; *Range* = 0–3) consistent with low risk (100%), while C2 participants reported a slightly higher number (*M* = 0.71; *SD* = 1.18; *Range* = 0–4), distributed between low (96.40%) and medium risk levels (3.60%).

**Figure 2:**
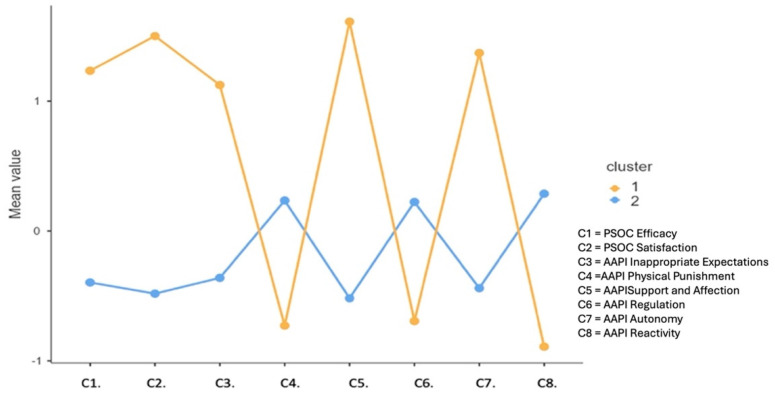
Average levels of variables characterizing parental behaviors by cluster.

Regarding the mental health level of the participants, in C1 the scores ranged from 33.00 to 62.00 points (*M* = 44.22; *SD* = 8.81), and in C2 the scores ranged from 32.00 to 64.00 (*M* = 48.07; *SD* = 8.77). The results also revealed that, in terms of the evaluation of children’s quality of life, C1 participants (*M* = 3.70; *SD* = 0.38) reported slightly lower levels than those in C2 (*M* = 3.86; *SD* = 0.28). Finally, as shown in Figure 2 and Table 3, there were differences in the characteristics of family functioning between the two clusters.

Regarding perceived parental competence, there were significant differences of moderate magnitude, with C1 participants showing higher gains in both efficacy (*Z* = −3.98; *p* < 0.001; *r* = 0.65) and satisfaction (*Z* = −4.32; *p* < 0.001; *r* = 0.71; *M*_Efficacy_ = 1.02; *SD*_Efficacy_ = 0.37; *M*_Satisfaction_ = 1.35; *SD*_Satisfaction_ = 0.62), compared to C2 (*M*_Efficacy_ = 0.23; *SD*_Efficacy_ = 0.33; *M*_Satisfaction_ = 0.06; *SD*_Satisfaction_ = 0.17). Regarding inappropriate expectations, both groups showed a statistically significant increase of moderate magnitude (*Z* = −3.18; *p* < 0.001; *r* = 0.52), but the increase was greater in C1 (*M* = 1.30; *SD* = 1.11) compared to C2 (*M* = 0.13; *SD* = 0.34). In terms of physical punishment, although the difference was marginally significant with low magnitude (*Z* = −1.70; *p* = 0.088; *r* = 0.28), the C1 participants showed a slight decrease in their average values (*M* = −0.56; *SD* = 1.01), while the C2 participants maintained their results (*M* = 0.02; *SD* = 0.29).

Regarding support and affection, there was a statistically significant difference of high magnitude (*Z* = −4.77; *p* < 0.001; *r* = 0.78), with C1 showing a higher result than C2 (*M*_C1_ = 1.18; *SD*_C1_ = 0.35; *M*_C2_ = 0.06; *SD*_C2_ = 0.12). In terms of regulation (*Z* = −2.51; *p* = 0.012; *r* = 0.41), C2 showed a smaller decrease (*M*_C1_ = −2.04; *SD*_C1_ = 0.51; *M*_C2_ = −1.29; *SD*_C2_ = 0.83), while C1 reported greater gains in autonomy (*Z* = −4.59; *p* < 0.001; *r* = 0.75; *M*_C1_ = 0.93; *SD*_C1_ = 0.52; *M*_C2_ = 0.04; *SD*_C2_ = 0.21) and a greater decrease in reactivity (*Z* = −2.51; *p* = 0.012; *r* = 0.41; *M*_C1_ = −0.62; *SD*_C1_ = 0.83; *M*_C2_ = −0.06; *SD*_C2_ = 0.13). All the differences were statistically significant, with moderate to high magnitudes.

In summary, the C1 participants overall reported higher gains on most of the evaluated dimensions (i.e., efficacy, satisfaction, inappropriate expectations, affection and support, and reactivity), while the C2 participants revealed changes only per the dimensions of physical punishment, regulation, and reactivity. To better interpret these results, a more detailed analysis of the values obtained by each cluster before the intervention was necessary (Table 4).

The comparison between C1 and C2 at the level of results obtained in the pre-test did not reveal significant differences or effects in any dimension. As Table 5 shows, the C2 participants attended slightly more program sessions (*M*_C2_ = 7.96; *SD*_C2_ = 2.56; *Range*_C2_ = 3–12) than those in C1 (*M*_C1_ = 7.78; *SD*_C1_ = 2.44; *Range*_C1_ = 3–10).

The comparison between pre-test and post-test revealed statistically significant results in almost all dimensions in both clusters. However, values from C1 reflected greater gains than those from C2. Notable were satisfaction (*Z*_C1_ = −2.67; *p*_C1_ = 0.008; *r*_C1_ = 0.47; *Z*_C2_ = −1.66; *p*_C2_ = 0.097; *r*_C2_ = 0.29; pre-test: *M*_C1_ = 3.57; *SD*_C1_ = 0.48; *M*_C2_ = 3.50; *SD*_C2_ = 0.50; post-test: *M*_C1_ = 4.91; *SD*_C1_ = 0.47; *M*_C2_ = 3.56; *SD*_C2_ = 0.46) and autonomy (*Z*_C1_ = −2.67; *p*_C1_ = 0.008; *r*_C1_ = 0.47; *Z*_C2_ = −1.65; *p*_C2_ = 0.098; *r*_C2_ = 0.29; pre-test: *M*_C1_ = 3.58; *SD*_C1_ = 0.42; *M*_C2_ = 3.50; *SD*_C2_ = 0.47; post-test: *M*_C1_ = 4.51; *SD*_C1_ = 0.39; *M*_C2_ = 3.54; *SD*_C2_ = 0.47), where the difference in C2 was nearly significant. Regarding reactivity, it was also in C1 where a greater decrease was observed (*Z*_C1_ = 2.40; *p*_C1_ = 0.027; *r*_C1_ = 0.39; *Z*_C2_ = −2.31; *p*_C2_ = 0.021; *r*_C2_ = 0.41; pre-test: *M*_C1_ = 2.42; *SD*_C1_ = 0.83; *M*_C2_ = 2.74; *SD*_C2_ = 0.79; post-test: *M*_C1_ = 1.80; *SD*_C1_ = 0.46; *M*_C2_ = 2.67; *SD*_C2_ = 0.78).

Although the difference was not significant in both clusters, C1 reported a greater increase in parental mental health (*Z*_C1_ = −1.87; *p*_C1_ = 0.236; *r*_C1_ = 0.33; *Z*_C2_ = −0.87; *p*_C2_ = 0.385; *r*_C2_ = 0.15; pre-test: *M*_C1_ = 44.22; *SD*_C1_ = 8.81; *M*_C2_ = 48.07; *SD*_C2_ = 8.77; post-test: *M*_C1_ = 47.78; *SD*_C1_ = 2.82; *M*_C2_ = 48.04; *SD*_C2_ = 8.29), while C2 participants presented higher average levels, with stability between assessed moments. Also noteworthy in terms of physical punishment was that, although not statistically significant, the average value was higher in C1, and it was also in this group where the decrease between moments was highest (*Z*_C1_ = −1.60; *p*_C1_ = 0.109; *r*_C1_ = 0.28; *Z*_C2_ = −0.57; *p*_C2_ = 0.609; *r*_C2_ = 0.09; pre-test: *M*_C1_ = 2.77; *SD*_C1_ = 0.63; *M*_C2_ = 2.71; *SD*_C2_ = 0.68; post-test: *M*_C1_ = 2.21; *SD*_C1_ = 0.67; *M*_C2_ = 2.73; *SD*_C2_ = 0.58).

## 4. Discussion

Considering the importance of positive parenting promotion programs in the development of parenting skills and the quality of life of children (e.g., Davies et al. [94]; Hidalgo et al. [95]), the present research aimed to study the changes promoted by the FAF intervention on parenting practices, perceived parental efficacy, and parents’ attitudes and beliefs. Therefore, a cluster analysis was conducted to identify groups of participants with similar gains, resulting in the definition of two groups/clusters (i.e., C1 and C2).

A preliminary analysis of the clusters (Figure 2), showing the differences between the assessment moments, indicated that both groups benefited from the program, with neither group experiencing deterioration due to the intervention. These results are similar to those of previous applications of the FAF, both in the European context [63,65,66] and in Peru [67].

It was also found that there was a greater difference in the evaluated dimensions in C1 compared to C2. To rule out the possibility that these differences already existed beforehand, a comparison of the two clusters before the intervention was conducted (Table 4), which revealed no pre-existing differences. Thus, the analysis of the characteristics of the participants in each cluster proceeded.

Regarding the sociodemographic characteristics and functional profile of the parents, the results did not highlight any differences in their profiles that would justify the different changes observed in the clusters. In terms of session attendance, considering that 14 sessions were held, no participants in either cluster attended all sessions, although the maximum attendance in C2 was slightly higher (12 sessions; C1 = 10 sessions). Past research on the implementation of parenting promotion programs in African contexts had shown that parents’ level of participation is quite high (e.g., Lachman et al. [70]), with low dropout rates, compared to developed countries [70,96]. However, it should be noted that parents’ levels of participation are an important predictor of the changes promoted by the intervention analyzed [3,7,10,97].

Concerning the dimensions associated with parenting, in C1, the results showed significant differences, and in most cases, of greater magnitude before and after the implementation of the FAF, per the dimensions of parental efficacy and satisfaction, inappropriate expectations, affection and support, and reactivity. In this sense, it appears that this group of participants, who started with lower pre-test values compared to C2, gained more benefits in terms of improved attitudes, regulation, and interaction with their children, feeling more effective and satisfied in this process.

According to the literature, when parents feel competent in their parenting tasks, using effective educational practices, and feeling satisfied with their parenting performance [9,49], they tend to use more parenting practices that include support and affection, trust in, and enhance children’s autonomy. As a result, they have less need to resort to regulation, use physical punishment, or find themselves in situations where they naturally feel more reactive. In summary, they are more inclined to engage in positive parenting practices rather than negative ones [6,16,17,98].

In turn, C2 showed a greater difference in regulation and reactivity, which appears to be a change largely focused on parenting practices and their implementation. Thus, the same activities can impact different parents differently [18]; however, the program resulted in changes across the various evaluated dimensions.

According to the standards of evidence developed by the Society for Prevention Research (SPR) for prevention programs and policies [99], the FAF can be considered an effective intervention because it was tested in at least two rigorous trials with defined samples from a specific population, using psychometrically sound measures and objective data collection procedures, and employing rigorous statistical approaches appropriate to the study’s objective. The findings have demonstrated that the effects obtained were consistent and maintained at follow-up. Furthermore, this intervention was based on a manualized program, which included both an intervention group and a control group, and the results of the intervention emphasize the importance of parental programs in parenting skills [100].

Overall, the FAF, like other programs implemented in African contexts, helped to increase positive parenting practices (e.g., Lachman et al. [70,101]; Rose et al. [71]), particularly in terms of parent-child relationships, positive parenting practices, perceived parental efficacy and satisfaction [12], and child maltreatment [70,97,101]. However, past studies had also highlighted the importance of various stressors on parents’ and groups’ responses to the intervention (e.g., Shenderovich et al. [98]; Littell and Schuerman [99]; Farrelly and McLennan [102]), including economic, educational, social, and health barriers, among others [69,71,97,101], which can negatively affect parents’ parenting skills and the development and well-being of their children [103].

In the present research, neither group showed a significant change in reducing physical punishment. It is important to emphasize that sociodemographic (e.g., family income and education level) and cultural characteristics can be important predictors of such practices [69,71,101]. Maya et al. [67] found similar results in Peru. These authors suggest that the practice of physical punishment as a form of behavioral control is more accepted in Latin American culture and, therefore, a more difficult dimension to change.

Some prior studies conducted in African contexts had indicated that families at psychosocial risk tend to exhibit higher levels of physical punishment. Other studies conducted among African cultural groups (e.g., Breen et al. [104]; Lachman et al. [70]) had suggested that physical punishment is a normative and culturally accepted disciplinary strategy. Therefore, programs and initiatives in this area have tended to show more modest results (e.g., Cluver et al. [105]; Lachman et al. [69,70,101]).

### Limitations and Future Studies

While this study makes important contributions to the field, it also had several limitations, such as the small number of participants and the limited number of evaluated domains; the reliability of some measures used; and the focus solely on quantitative data, which restricted interpretation of the changes promoted by the FAF intervention. We recommend that future studies use previously adapted instruments to the context and employ mixed methods approaches. These approaches could involve returning the results to the parents and discussing them together, as well as including an assessment of the children’s behavior and other evaluation sources. It is always important to consider that interventions in such programs should be evidence-based and meet the quality criteria for these types of actions [95,106]. Furthermore, it is crucial to exhaustively examine the various predictors and differentiators (e.g., mediators, moderators) of the changes achieved (or not) promoted (e.g., Decker et al. [73]). Future studies might also utilize qualitative methodologies and include an evaluation of the children’s behavior, along with other assessment sources.

## 5. Conclusions

This study is an important contribution to promoting positive parenting programs, particularly in the African context. Past research had shown that such programs can have a positive impact on families, although their implementation must be subject to cultural adaptations. Our study, by analyzing the underlying profiles of these change processes, highlighted that some participants benefit more from the intervention than others, which is valuable information for professionals and researchers.

## Figures and Tables

**Table 1:** Optimal scores.

Validation Measures		Optimal Score	Method	Clusters
Internal	Connectivity	6.691	Diana	2
	Dunn Index	0.439	Diana	3
	Silhouette	0.525	Diana	2
Stability	APN	0.007	Hierarchical	2
	AD	1.389	Sota	3
	ADM	0.023	Hierarchical	2
	FOM	0.400	Sota	3

Notes. APN = Average proportion of non-overlap; AD = Average distance; ADM = Average distance between means; FOM = Figure of merit.

**Table 2:** Sociodemographic and psychosocial characteristics of participants by cluster.

Domains	Categories	Cluster 1 (*n* = 9)				Cluster 2 (*n* = 28)			
		*f*		*%*		*f*		*%*	
Assistance Group	1	0		0		1		3.60	
	2	1		11.10		10		35.70	
	3	8		88.90		8		28.60	
	4	0		0		9		32.10	
Education Level	Primary	5		55.50		53		53.50	
	Secondary	3		33.30		8		28.60	
	Higher education	1		11.10		5		17.90	
Family Type	Monoparental	3		33.30		5		17.90	
	Biparental	6		66.70		20		71.40	
Sex of the child	Female	3		33.30		14		50.00	
	Male	6		66.70		14		50.00	
		*M*	*SD*	*Min*	*Max*	*M*	*SD*	*Min*	*Max*
Age of the child		8.33	2.50	6.00	12.00	8.79	2.23	6.00	12.00
Mental health		44.22	8.81	33.00	62.00	48.07	8.77	32.00	64.00
Child Quality of Life		3.70	0.38	2.80	4.00	3.86	0.28	2.90	4.30

Note. *f* = frequency, % = percentage, *M* = Mean, *SD* = Standard Deviation, *Min* = Minimum, *Max* = Maximum.

**Table 3:** Effect of the intervention on parental behaviors and practices (difference between the average values of M1 and M2) by cluster.

Domains	Cluster 1 (*n* = 9)				Cluster 2 (*n* = 28)				*Z*	*p*	*r*
	*M*	*SD*	*Min*	*Max*	*M*	*SD*	*Min*	*Max*			
Efficacy	1.02	0.37	0.43	1.57	0.23	0.33	−0.57	1.14	−3.98	<0.001	0.65
Satisfaction	1.35	0.62	0.22	2.22	0.06	0.17	−0.22	0.44	−4.32	<0.001	0.71
Inap. Exp.	1.30	1.11	−0.14	2.86	0.13	0.34	−0.29	1.29	−3.18	0.001	0.52
Physical Punishment	−0.56	1.01	−2.00	1.09	0.02	0.29	−0.64	0.91	−1.70	0.088	0.28
Affection and Support	1.18	0.35	0.80	2.00	0.06	0.12	−0.20	0.40	−4.77	<0.001	0.78
Regulation	−2.04	0.51	−2.80	−1.20	−1.29	0.83	−3.00	0.20	−2.51	0.012	0.41
Autonomy	0.93	0.52	0.20	1.80	0.04	0.21	−0.80	0.40	−4.59	<0.001	0.75
Reactivity	−0.62	0.83	−2.40	0.00	−0.06	0.13	−0.40	0.20	−2.51	0.012	0.41

Note. Inap. Exp. = Inappropriate Expectations; *M* = Mean; *SD* = Standard Deviation; *Min* = Minimum; *Max* = Maximum; *Z* = test statistic; *p* = significance; *r* = effect size.

**Table 4:** Comparison between clusters in the pre-test.

Dimensions	C1		C2		*Z*	*p*	*r*
	*M*	*SD*	*M*	*SD*			
Efficacy	4.32	0.25	4.31	0.49	−0.27	0.789	0.04
Satisfaction	3.57	0.48	3.50	0.50	−0.28	0.776	0.05
Quality of Life	3.70	0.38	3.86	0.28	−1.33	0.183	0.22
Parental Mental Health	44.22	8.81	48.07	8.77	−1.45	0.146	0.24
Inappropriate Expectations	2.40	0.71	2.15	0.66	−1.14	0.255	0.19
Physical Punishment	2.77	0.63	2.71	0.68	−0.21	0.831	0.04
Affection and Support	3.76	0.36	3.95	0.43	−1.13	0.259	−0.19
Regulation	3.84	0.51	3.96	0.30	−0.72	0.475	−0.12
Autonomy	3.58	0.42	3.50	0.47	−0.41	0.680	−0.07
Reactivity	2.42	0.83	2.74	0.79	−1.16	0.248	−0.19

Note. *M* = Mean; *SD* = Standard deviation; *Z* = Test statistic; *p* = Significance; *r* = Effect size.

**Table 5:** Comparison between pre-test and post-test in both clusters.

Domains	Cluster 1 (*n* = 9)								*Z*	*p*	*r*	Cluster 2 (*n* = 28)								*Z*	*p*	*r*
	*Pre*				*Post*							*Pre*				*Post*						
	*M*	*SD*	*Min*	*Max*	*M*	*SD*	*Min*	*Max*				*M*	*SD*	*Min*	*Max*	*M*	*SD*	*Min*	*Max*			
No. of sessions	-	-	-	-	7.78	2.44	3.00	10.00	-	-	-	-	-	-	-	7.96	2.56	3.00	12.00	-	-	-
Efficacy	4.32	0.25	4.00	4.71	5.33	0.29	4.86	5.71	−2.67	0.008	0.47	4.31	0.49	3.14	5.43	4.55	0.41	3.57	5.29	−3.17	0.002	0.56
Satisfaction	3.57	0.48	3.00	4.44	4.91	0.47	4.11	5.56	−2.67	0.008	0.47	3.50	0.50	2.67	4.67	3.56	0.46	2.67	4.78	−1.66	0.097	0.29
Quality of Life	3.70	0.38	2.80	4.00	4.52	0.28	4.20	5.00	2.67	0.008	0.47	3.86	0.28	2.90	4.30	3.96	0.32	2.80	4.50	−3.03	0.002	0.54
Parental Mental Health	44.22	8.81	33.00	62.00	47.78	2.82	44.00	53.00	−1.87	0.236	0.33	48.07	8.77	32.00	64.00	48.04	8.29	33.00	64.00	−0.87	0.385	0.15
Inappropriate Expectations	2.40	0.71	1.43	3.29	3.70	0.49	3.00	4.43	−2.94	0.013	0.52	2.15	0.66	1.14	3.29	2.29	0.71	1.14	3.57	−2.20	0.028	0.39
Physical Punishment	2.77	0.63	2.00	3.55	2.21	0.67	1.18	3.18	−1.60	0.109	0.28	2.71	0.68	1.73	4.09	2.73	0.58	1.73	4.00	−0.51	0.609	0.09
Affection and Support	3.76	0.36	3.00	4.20	4.93	0.14	4.60	5.00	−2.69	0.007	0.48	3.95	0.43	3.00	5.00	4.01	0.38	3.00	4.80	−2.50	0.013	0.44
Regulation	3.84	0.51	3.00	4.60	4.87	0.22	4.40	5.00	−2.68	0.007	0.47	3.96	0.30	3.20	4.80	4.01	0.30	3.40	4.80	−2.83	0.005	0.50
Autonomy	3.58	0.42	2.80	4.00	4.51	0.39	4.00	5.00	−2.67	0.008	0.47	3.50	0.47	2.40	4.20	3.54	0.47	2.40	4.20	−1.65	0.098	0.29
Reactivity	2.42	0.83	1.40	3.60	1.80	0.46	1.20	2.40	−2.21	0.027	0.39	2.74	0.79	1.00	3.80	2.67	0.78	1.00	3.80	−2.31	0.021	0.41

Note. *M* = Mean; *SD* = Standard deviation; *Z* = Test statistic; *p* = Significance; *r* = Effect size.

## Data Availability

The data can be made available for consultation from the corresponding author upon request. The data are not publicly available due to privacy and ethical restrictions.
